# Corticotropin-Releasing Factor: An Ancient Peptide Family Related to the Secretin Peptide Superfamily

**DOI:** 10.3389/fendo.2020.00529

**Published:** 2020-08-27

**Authors:** Ola M. Michalec, Belinda S. W. Chang, Nathan R. Lovejoy, David A. Lovejoy

**Affiliations:** ^1^Department of Cell and Systems Biology, University of Toronto - St. George Campus, Toronto, ON, Canada; ^2^Department of Biological Sciences, University of Toronto Scarborough, Toronto, ON, Canada

**Keywords:** secretin superfamily, TCAP, teneurin, adhesion GPCRs, evolution, metabolism

## Abstract

Corticotropin-releasing factor (CRF) is the hypothalamic releasing peptide that regulates the hypothalamic-pituitary-adrenal/inter-renal (HPA/I) axis in vertebrates. Over the last 25 years, there has been considerable discussion on its paralogs genes, urotensin-I/urocortin-1, and urocortins-2 and-3 and their subsequent role in the vertebrate stress response. Phylogenetically, the CRF family of peptides also belong to the diverse assemblage of Secretin- and Calcitonin-based peptides as evidenced by comparative-based studies of both their ligand and G-protein-coupled receptor (GPCR) structures. Despite this, the common origin of this large assemblage of peptides has not been ascertained. An unusual peptide, teneurin-C-terminal associated peptide (TCAP), reported in 2004, comprises the distal extracellular tip of the teneurin transmembrane proteins. Further studies indicated that this teneurin region binds to the latrophilin family of GPCRs. Initially thought to be a member of the Secretin GPCR family, evidence indicates that the latrophilins are a member of the Adhesion family of GPCRs and are related to the common ancestor of both Adhesion and Secretin GPCR families. In this study, we posit that TCAP may be a distantly related ancestor of the CRF-Calcitonin-Secretin peptide family and evolved near the base of metazoan phylogeny.

## Introduction

Corticotropin-releasing factor (CRF) is the critical hypothalamic releasing factor that regulates the hypothalamus-pituitary-adrenal/inter-renal (HPA/1) axis in vertebrates, yet after some 40 years after its discovery, numerous questions still exist regarding when, why, and how this peptide evolved. We hypothesize that due to the high level of primary structure similarity among CRF paralogs and related peptide lineages (e.g., calcitonin, secretin) there was likely an ancestor peptide common to this cluster. We further suggest that the “teneurin C-terminal associated peptides” (TCAP) represent an extant candidate lineage related to the hypothetical common ancestor.

The discovery of CRF in the early 1980s (1) occurred about the same time as the discovery of other peptides of similar structure [sauvagine (2); urotensin-I (3)]. Later, Vale and his laboratory characterized a mammalian version of sauvagine/urotensin-I in rat brain that they termed, urocortin (4). Further phylogenetic studies suggested that mammalian urocortin, amphibian sauvagine, and fish urotensin-I were orthologs of the same gene (5). In 2001, the structures of two novel related peptides were reported by the Vale laboratory who named the peptides, urocortin 2 and 3 (6, 7) and by Hsu and Hsieh (8) who termed the peptides as “stresscopin” and “stresscopin-related peptide.” These novel CRF family homologs were subsequently established to be a separate paralogs lineage of CRF and the urotensin-I/sauvagine/urocortin grouping (5, 9–13). In parallel to studies of vertebrate CRF isoforms, the presence of related peptides were reported in insects and arthropods (12, 14–16). Therefore, the high degree of structural similarity among CRF-like peptides in both deuterostome (e.g., chordates) and protostomes (e.g., arthropods) indicated that an ancestral peptide with CRF family primary structure attributes was present before the bifurcation of these metazoan lineages. Importantly, this ancestral peptide appeared to exist in a physiologically mature form indicative of a distant lineage that likely radiated as other ancestral peptides with distinct but overlapping functions. The identity of these hypothetical ancestral peptides has remained elusive, however, it is plausible that these lineages led to the evolution and expansion of the secretin and calcitonin family of peptides (11, 12).

The Secretin superfamily of peptides is a diverse assemblage of peptide lineages with overlapping functions utilizing structurally related receptors. The nomenclature describing the phylogeny of the secretin grouping of peptides and receptors is confusing. In order to clarify this, we have used the term “secretin family” to denote those peptides that are thought to be part of a direct monophyletic clade (e.g., secretin, PACAP, VIP, and glucagon paralogs). For the inclusion of the wider group which include CRF and calcitonin, we have referred to this as the “Secretin superfamily.” Due, in part, to the similarity and structural conservation of their cognate receptors, the Secretin family G-Protein Coupled Receptors (GPCR) was defined as a distinct clade (17). The Secretin superfamily of peptides is one of the five main families of ligands that bind to GPCRs. The GPCRs have most recently been classified into five main families using the GRAFS system; Glutamate (G), Rhodopsin (R), Adhesion (A), Frizzled/Taste2 (F), and Secretin (S) (17). Notably, both CRF and calcitonin receptors are included within the Secretion GPCR family. Among these, Adhesion and Secretin GPCRs are the most evolutionarily ancient (18). Adhesion GPCRs have a characteristically long N-terminus rich in serine and threonine residues whereas Secretin GPCRs have a characteristic hormone-binding domain (HBD) in their N-terminal region (18). Secretin-related receptors form a single monophyletic clade that derived from the Adhesion GPCRs (18, 19). Adhesion GPCR genes have been identified in choanoflagellate and sea anemone genomes but Secretin GPCR genes have not suggesting that Adhesion GPCRs are more evolutionarily ancient than Secretin GPCRs (18). Interestingly, some derived phylogenetically younger Adhesion GPCR members possess an HBD with highly conserved amino acid sequences and similar splice site motifs as Secretin GPCRs. These observations led, in part, to the hypothesis that the Secretin GPCR clade was derived from an offshoot of the Adhesion GPCR lineage. However, although the data linking the Adhesion and Secretin superfamilies were compelling, evidence of a structurally related peptide ligand linking the two receptor clades was lacking.

One such lineage of Adhesion GPCRs that does possess a HBD with similar structural motifs to Secretin GPCRs are the latrophilins (LPHN) or ADGRL (Adhesion G-protein coupled receptors, subfamily L). It was originally considered a new type of Secretin GPCR, due to its characteristic HBD, but has now been re-classified as an Adhesion GPCR (17). The first identified ligand for ADGRL was α-latrotoxin, a peptide component of black widow spider toxin venom that specifically targets vertebrates (20) and shares major sequence similarity with other Secretin superfamily ligands (21). The data suggest that these peptides have a common origin. Although, α-latrotoxin was an exogenous ligand, the high affinity binding of this soluble peptide to ADGRL indicated that this receptor had the potential to bind and be activated by an endogenous peptide similar to the structure of α-latrotoxin. The search for this theoretical ligand led to the identification of the teneurin transmembrane proteins as a likely suspect.

Several recent studies established that the distal region of the extracellular domain of the teneurin transmembrane proteins binds ADGRL with high affinity and activates the receptor. Silva et al. (22) first discovered that teneurin-2, expressed on post-synaptic dendritic branches, binds LPHN-1 expressed on pre-synaptic nerve terminals to form a trans-synaptic complex. Similar trans-cellular interactions were observed between teneurins-2 and 4 and all three LPHNs (23) and between teneurin-1 and LPHN-3 (24). A C-terminal fragment of teneurin-2, named Lasso, triggered an increase in cytosolic Ca^2+^ in Nb2a cells overexpressing LPHN-1 and in pre-synaptic nerve terminals of hippocampal cells (22). This distal region of the teneurin extracellular domain contains a peptide-like sequence termed “teneurin C-terminal associated peptide” (TCAP). The TCAPs are a family of four bioactive peptides that are 40–41 amino acids in length and are located at the C-terminus of each of the teneurin transmembrane proteins (25, 26). TCAPs possess a cleavage motif at the N-terminus and an amidation motif at the C-terminus (27) and may be autolytically cleaved from teneurins upon binding with LPHN (28, 29). TCAP shares about 20% sequence similarity with CRF and calcitonin, members of the Secretin superfamily of ligands, suggesting a common evolutionary origin (27). Moreover, our laboratory has recently identified that teneurin C-terminal associated peptide (TCAP)-1 is likely an endogenous ligand that interacts with the HBD of LPHN (30).

Therefore, as TCAP binds to an Adhesion GPCR and shares sequence similarity to CRF and calcitonin, ligands that bind to Secretin GPCR receptors that are classified as being most closely related to ancestral Adhesion GPCRs, this prompted the investigation of TCAP as a progenitor of the Secretin superfamily. The hypothesis that the teneurin-TCAP system is an ancient system that arose prior to the emergence of the Metazoa as a result of a horizontal gene transfer (HGT) event from a prokaryote to a choanoflagellate ancestor has previously been raised (31–33). However, the TCAP family has not been previously examined. Thus, TCAP may be associated with an early evolving lineage of peptides that is a sister lineage to the CRF, calcitonin, and secretin families of peptides (11, 34). We therefore examined the phylogenetic relationships of these peptides using TCAP as an outgroup.

## Materials and Methods

### Collection of Sequences

Peptide sequences of Secretin GPCR ligands, including CRF, calcitonin and secretin families, and Adhesion GPCR ligands, including TCAP 1–4, as well as reference groups including neuropeptide Y (NPY) and insulin were collected among a range of extant protostomes and deuterostomes, using the GenBank genome sequence analysis program on the NCBI website. The peptides were organized by organism, phylum, class, and order and were tabulated and their accession numbers were recorded (Table 1). Sequences were divided into pre-propeptides (or propeptides for TCAP) and mature peptides, after which were imported to MEGA 6.0 for analysis (38). Downloaded from http://www.megasoftware.net/.

**Table 1:** List of Sequences used to construct phylogenetic trees.

**Hormone**	**Organism**	**Phylum**	**Class**	**Order**	**Accession number**
**CRF FAMILY**					
CRF	*Xenopus laevis* (African clawed frog)	Chordata	Amphibia	Anura	NM_001172210
CRF	Rana sylvatica (Wood frog)	Chordata	Amphibia	Anura	HQ630608
CRF	*Gallus gallus* (Chicken)	Chordata	Aves	Galliformes	NM_001123031
CRF	*Columba livia* (Rock pigeon)	Chordata	Aves	Columbiformes	XM_005506466
CRF	*Danio rerio* (Zebrafish)	Chordata	Actinopterygii	Cypriniformes	JN859047
CRF	*Salmo salar* (Atlantic salmon)	Chordata	Actinopterygii	Salmoniformes	NM_001141590
CRF	*Mus musculus* (House mouse)	Chordata	Mammalia	Rodentia	NM_205769 XM_357335
CRF	*Canis lupus familiaris* (Dog)	Chordata	Mammalia	Carnivora	NM_001014278 XM_544106
CRF	*Petromyzon marinus* (Sea lamprey)	Chordata	Cephalaspidomorphi	Petromyzontiformes	Endsin et al. (35)
CRF	*Latimeria chalumnae* (Coelacanth)	Chordata	Sarcopterygii	Coelacanthiformes	XM_006009123
CRF	*Chrysemys picta bellii* (Western painted turtle)	Chordata	Reptilia	Testudines	XM_005288085
CRF	*Asterias rubens* (Common starfish)	Echinodermata	Asteroidea	Forcipulatida	Semmens et al. (36)
CRF	*Ciona intestinalis* (Sea tunicate)	Urochordata	Ascidiacea	Enterogona	Lovejoy and Barsyte-Lovejoy, (37)
TCN	*Oryzias latipes* (Japanese rice fish)	Chordata	Actinopterygii	Beloniformes	BAR90710.1
SVG	*Pachymedusa dacnicolor* (Mexican tree frog)	Chordata	Amphibia	Anura	FR846380
SVG	*Phyllomedusa sauvagii*	Chordata	Amphibia	Anura	AAY21509.1
UCN	*Pseudopodoces humilis* (Tibetan ground-tit)	Chordata	Aves	Passeriformes	XM_005532743
UCN	*Melopsittacus undulatus* (Budgerigar)	Chordata	Aves	Psittaciformes	XM_005140144
UCN	*Mus musculus* (House mouse)	Chordata	Mammalia	Rodentia	NM_021290
UCN	*Canis lupus familiaris* (Dog)	Chordata	Mammalia	Carnivora	XM_848667
UCN	*Alligator mississippiensis* (American alligator)	Chordata	Reptilia	Crocodylia	XM_006273603
UCN2	*Sus scrofa* (Pig)	Chordata	Mammalia	Artiodactyla	XM_003132185
UCN2	*Bos taurus* (Cattle)	Chordata	Mammalia	Artiodactyla	XM_005223016
UCN2	*Mus musculus* (House mouse)	Chordata	Mammalia	Rodentia	NM_145077 XM_910603
UCN2	*Canis lupus familiaris* (Dog)	Chordata	Mammalia	Carnivora	XM_005632587
UCN2	*Oryzias latipes* (Japanese rice fish)	Chordata	Actinopterygii	Beloniformes	NP_001121991.1
UCN3	*Anas platyrhynchos* (Mallard)	Chordata	Aves	Anseriformes	XM_005014551
UCN3	*Mus musculus* (House mouse)	Chordata	Mammalia	Rodentia	NM_031250
UCN3	*Bos taurus* (Cattle)	Chordata	Mammalia	Artiodactyla	NM_001076527
UCN3	*Alligator sinensis* (Chinese alligator)	Chordata	Reptilia	Crocodylia	XM_006035307
UCN3	*Petromyzon marinus* (Sea lamprey)	Chordata	Cephalaspidomorphi	Petromyzontiformes	Endsin et al. (35)
UCN3	*Oryzias latipes* (Japanese rice fish)	Chordata	Actinopterygii	Beloniformes	NP_001121992.1
UI	*Oncorhynchus mykiss* (Rainbow trout)	Chordata	Actinopterygii	Salmoniformes	NM_001124343
UI	*Danio rerio* (Zebrafish)	Chordata	Actinopterygii	Cypriniformes	NM_001030180 XM_687090
UI	*Carassius auratus* (Goldfish)	Chordata	Actinopterygii	Cypriniformes	AF129115
UI	*Petromyzon marinus* (Sea lamprey)	Chordata	Cephalaspidomorphi	Petromyzontiformes	Endsin et al. (35)
DH 31	*Drosophila melanogaster* (Fruit fly)	Arthropoda	Insecta	Diptera	NM_078790
DH 40	*Bombyx mori* (Domestoc silkworm)	Arthropoda	Insecta	Lepidoptera	AB298934
DH 31	*Rhodnius prolixus* (Assassin bug)	Arthropoda	Insecta	Hemiptera	HM030716
DH	*Balanus amphitrite* (Barnacle)	Arthropoda	Maxillopoda	Sessilia	JQ864196
**CALCITONIN FAMILY**					
CALC	*Gallus gallus* (Chicken)	Chordata	Aves	Galliformes	X03012
CALC	*Bos taurus* (Cattle)	Chordata	Mammalia	Artiodactyla	AB462435
CALC	*Mus musculus* (House mouse)	Chordata	Mammalia	Rodentia	X97991
CALC	*Alligator sinensis* (Chinese alligator)	Chordata	Reptilia	Crocodylia	XM_006018232
CALC	*Chrysemys picta bellii* (Western painted turtle)	Chordata	Reptilia	Testudines	XM_005303304
CALC	*Ciona intestinalis* (Sea tunicate)	Urochordata	Ascidiacea	Enterogona	AB485672
CALC	*Equus caballus* (Horse)	Chordata	Mammalia	Perissodactyla	AF249307
CALC	*Chinchilla lanigera* (Long-tailed chinchilla)	Chordata	Mammalia	Rodentia	XP_005380394.1
CALC	Spermophilus tridecemlineatus (Squirrel)	Chordata	Mammalia	Rodentia	XM_005326775
CGRP1	*Gallus gallus* (Chicken)	Chordata	Aves	Galliformes	NM_001113708
CGRP1	*Danio rerio* (Zebrafish)	Chordata	Actinopterygii	Cypriniformes	NM_001002471
CGRP1	*Salmo salar* (Atlantic salmon)	Chordata	Actinopterygii	Salmoniformes	NM_001146580
CGRP1	*Bos taurus* (Cattle)	Chordata	Mammalia	Artiodactyla	NM_001076340
CGRP1	*Canis lupus familiaris* (Dog)	Chordata	Mammalia	Carnivora	NM_001003266
CGRP2	*Callorhinchus milii* (Elelphant shark)	Chordata	Chondrichthyes	Chimaeriformes	XM_007887527
CGRP2	*Bos taurus* (Cattle)	Chordata	Mammalia	Artiodactyla	NM_001134662
CGRP2	*Canis lupus familiaris* (Dog)	Chordata	Mammalia	Carnivora	NM_001002948
CGRP2	*Mus musculus* (House mouse)	Chordata	Mammalia	Rodentia	NM_054084
CGRP2	*Sus scrofa* (Pig)	Chordata	Mammalia	Artiodactyla	NM_001102473
CGRP2	*Oryzias latipes* (Japanese rice fish)	Chordata	Actinopterygii	Beloniformes	NM_001104894
Amylin	*Columba livia* (Rock pigeon)	Chordata	Aves	Columbiformes	XM_005504095
Amylin	*Gallus gallus* (Chicken)	Chordata	Aves	Galliformes	NM_205397
Amylin	*Carassius auratus* (Goldfish)	Chordata	Actinopterygii	Cypriniformes	EU000530
Amylin	*Latimeria chalumnae* (Coelacanth)	Chordata	Sarcopterygii	Coelacanthiformes	XM_005998862
Amylin	*Bos taurus* (Cattle)	Chordata	Mammalia	Artiodactyla	NM_001195038
Amylin	*Mus musculus* (House mouse)	Chordata	Mammalia	Rodentia	NM_010491
Amylin	*Sus scrofa* (Pig)	Chordata	Mammalia	Artiodactyla	XM_003126437
Amylin	*Alligator mississippiensis* (American alligator)	Chordata	Reptilia	Crocodylia	XM_006270879
ADM	*Xenopus tropicalis* (Western clawed frog)	Chordata	Amphibia	Anura	XM_002936741
ADM	*Columba livia* (Rock pigeon)	Chordata	Aves	Columbiformes	XM_005499377
ADM	*Carassius auratus* (Goldfish)	Chordata	Actinopterygii	Cypriniformes	EU000533
ADM	*Latimeria chalumnae* (Coelacanth)	Chordata	Sarcopterygii	Coelacanthiformes	XM_005989689
ADM	*Sus scrofa* (Pig)	Chordata	Mammalia	Artiodactyla	D14875
ADM	*Bos taurus* (Cattle)	Chordata	Mammalia	Artiodactyla	NM_173888
ADM	*Canis lupus familiaris* (Dog)	Chordata	Mammalia	Carnivora	AB191461
ADM	*Mus musculus* (House mouse)	Chordata	Mammalia	Rodentia	NM_009627
ADM2	*Xenopus tropicalis* (Western clawed frog)	Chordata	Amphibia	Anura	XM_002939371
ADM2	*Gallus gallus* (Chicken)	Chordata	Aves	Galliformes	XM_004937395
ADM2	*Latimeria chalumnae* (Coelacanth)	Chordata	Sarcopterygii	Coelacanthiformes	XM_006013419
ADM2	*Poecilia formosa* (Amazon molly)	Chordata	Actinopterygii	Cyprinodontiformes	XM_007545759
ADM2	*Canis lupus familiaris* (Dog)	Chordata	Mammalia	Carnivora	XM_843399
ADM2	*Mus musculus* (House mouse)	Chordata	Mammalia	Rodentia	AB121035
ADM2	*Alligator mississippiensis* (American alligator)	Chordata	Reptilia	Crocodylia	XM_006275255
**SECRETIN FAMILY**					
GHRH	*Xenopus laevis* (African clawed frog)	Chordata	Amphibia	Anura	NM_001096728
GHRH	*Gallus gallus* (Chicken)	Chordata	Aves	Galliformes	NM_001040464
GHRH	*Callorhinchus milii* (Elelphant shark)	Chordata	Chondrichthyes	Chimaeriformes	XM_007885752
GHRH	*Danio rerio* (Zebrafish)	Chordata	Actinopterygii	Cypriniformes	NM_001080092
GHRH	*Bos taurus* (Cattle)	Chordata	Mammalia	Artiodactyla	NM_178325
GHRH	*Canis lupus familiaris* (Dog)	Chordata	Mammalia	Carnivora	NM_001290112
GHRH	*Mus musculus* (House mouse)	Chordata	Mammalia	Rodentia	NM_010285
GHRH	*Sus scrofa* (Pig)	Chordata	Mammalia	Artiodactyla	NM_001195118
GHRH	*Anolis carolinensis* (Green anole)	Chordata	Reptilia	Squamata	XM_003225298
GHRH	*Chrysemys picta bellii* (Western painted turtle)	Chordata	Reptilia	Testudines	XM_005295322
GIP	*Xenopus laevis* (African clawed frog)	Chordata	Amphibia	Anura	NM_001097922
GIP	*Gallus gallus* (Chicken)	Chordata	Aves	Galliformes	NM_001080104
GIP	*Danio rerio* (Zebrafish)	Chordata	Actinopterygii	Cypriniformes	NM_001080059
GIP	*Bos taurus* (Cattle)	Chordata	Mammalia	Artiodactyla	NM_001166605
GIP	*Mus musculus* (House mouse)	Chordata	Mammalia	Rodentia	NM_008119
GIP	*Sus scrofa* (Pig)	Chordata	Mammalia	Artiodactyla	NM_001287408
GIP	*Alligator mississippiensis* (American alligator)	Chordata	Reptilia	Crocodylia	XM_006277905
GIP	*Chelonia mydas* (Green sea turtle)	Chordata	Reptilia	Testudines	XM_007061917
GCG	*Gallus gallus* (Chicken)	Chordata	Aves	Galliformes	NM_205260
GCG	*Callorhinchus milii* (Elelphant shark)	Chordata	Chondrichthyes	Chimaeriformes	XM_007889848
GCG	*Bos taurus* (Cattle)	Chordata	Mammalia	Artiodactyla	NM_173916
GCG	*Sus scrofa* (Pig)	Chordata	Mammalia	Artiodactyla	NM_214324
GCG	*Ovis aries* (Sheep)	Chordata	Mammalia	Cetartiodactyla	XM_004004659
GCG	*Alligator mississippiensis* (American alligator)	Chordata	Reptilia	Crocodylia	XM_006277994
GCG	*Poecilia formosa* (Amazon molly)	Chordata	Actinopterygii	Cyprinodontiformes	XM_007546594
GCG	*Latimeria chalumnae* (Coelacanth)	Chordata	Sarcopterygii	Coelacanthiformes	XM_006004345
PACAP	*Xenopus laevis* (African clawed frog)	Chordata	Amphibia	Anura	AF187877
PACAP	*Gallus gallus* (Chicken)	Chordata	Aves	Galliformes	AY956323
PACAP	*Ctenopharyngodon idella* (Grass carp)	Chordata	Actinopterygii	Cypriniformes	EF592488
PACAP	*Bos taurus* (Cattle)	Chordata	Mammalia	Artiodactyla	AY924308
SCT	*Gallus gallus* (Chicken)	Chordata	Aves	Galliformes	NM_001024833
SCT	*Taeniopygia guttata* (Zebra finch)	Chordata	Aves	Passeriformes	NM_001256233
SCT	*Mus musculus* (House mouse)	Chordata	Mammalia	Rodentia	X73580
SCT	*Sus scrofa* (Pig)	Chordata	Mammalia	Artiodactyla	XM_003122391
SCT	*Equus caballus* (Horse)	Chordata	Mammalia	Perissodactyla	XM_003362642
SCT	*Chelonia mydas* (Green sea turtle)	Chordata	Reptilia	Testudines	XM_007060911
VIP	*Xenopus laevis* (African clawed frog)	Chordata	Amphibia	Anura	NM_001085714
VIP	*Columba livia* (Rock pigeon)	Chordata	Aves	Columbiformes	XM_005507654
VIP	*Gallus gallus* (Chicken)	Chordata	Aves	Galliformes	NM_205366
VIP	*Danio rerio* (Zebrafish)	Chordata	Actinopterygii	Cypriniformes	NM_001114553
VIP	*Bos taurus* (Cattle)	Chordata	Mammalia	Artiodactyla	AF503910
VIP	*Canis lupus familiaris* (Dog)	Chordata	Mammalia	Carnivora	XM_005615524
VIP	*Mus musculus* (House mouse)	Chordata	Mammalia	Rodentia	NM_011702
VIP	*Sus scrofa* (Pig)	Chordata	Mammalia	Artiodactyla	NM_001195233
VIP	*Alligator mississippiensis* (American alligator)	Chordata	Reptilia	Crocodylia	XM_006265239
**OTHER**					
NPY	*Mus musculus* (House mouse)	Chordata	Mammalia	Rodentia	EDK98613.1
NPY	*Danio rerio* (Zebrafish)	Chordata	Actinopterygii	Cypriniformes	AAI62071.1
NPY	*Carassius auratus* (Goldfish)	Chordata	Actinopterygii	Cypriniformes	AAA49186.1
NPY	*Rattus norvegicus* (Norway rat)	Chordata	Mammalia	Rodentia	NP_036746.1
NPY	*Gallus gallus* (Chicken)	Chordata	Aves	Galliformes	NP_990804.1
NPY	*Xenopus laevis* (African clawed frog)	Chordata	Amphibia	Anura	AAH80115.1
NPY	*Ovis aries* (Sheep)	Chordata	Mammalia	Cetartiodactyla	NP_001009452.1
NPY	*Bos taurus* (Cattle)	Chordata	Mammalia	Artiodactyla	ACH61954.1
NPY	*Homo sapiens* (Human)	Chordata	Mammalia	Primate	NP_000896.1
NPY	*Columba livia* (Rock pigeon)	Chordata	Aves	Columbiformes	NP_001269740.1
NPY	*Pseudopodoces humilis* (Ground tit)	Chordata	Aves	Passeriformes	XP_005518939.1
NPY	*Callorhinchus milii* (Elelphant shark)	Chordata	Chondrichthyes	Chimaeriformes	ACF22970.1
NPY	*Chrysemys picta bellii* (Western painted turtle)	Chordata	Reptilia	Testudines	XP_005290923.1
INS	*Ciona intestinalis* (Sea tunicate)	Urochordata	Ascidiacea	Enterogona	NP_001123204.1
INS	*Mus musculus* (House mouse)	Chordata	Mammalia	Rodentia	ABF48502.1
INS	*Rattus norvegicus* (Norway rat)	Chordata	Mammalia	Rodentia	AAA41439.1
INS	*Ictidomys tridecemlineatus* (Ground squirrel)	Chordata	Mammalia	Rodentia	AAK72558.1
INS	*Ovis aries* (Sheep)	Chordata	Mammalia	Cetartiodactyla	AAB60625.1
INS	*Gallus gallus* (Chicken)	Chordata	Aves	Galliformes	NP_990553.1
INS	*Columba livia* (Rock pigeon)	Chordata	Aves	Columbiformes	EMC88047.1
INS	*Pseudopodoces humilis* (Ground tit)	Chordata	Aves	Passeriformes	XP_005522396.1
INS	*Callorhinchus milii* (Elelphant shark)	Chordata	Chondrichthyes	Chimaeriformes	XP_007902984.1
INS	*Danio rerio* (Zebrafish)	Chordata	Actinopterygii	Cypriniformes	NP_571131.1
INS	*Carassius auratus* (Goldfish)	Chordata	Actinopterygii	Cypriniformes	ALO24192.1
INS	*Salmo salar* (Atlantic salmon)	Chordata	Actinopterygii	Salmoniformes	ACI69187.1
INS	*Latimeria chalumnae* (Coelacanth)	Chordata	Sarcopterygii	Coelacanthiformes	XP_006008147.1
INS	*Poecilia formosa* (Amazon molly)	Chordata	Actinopterygii	Cyprinodontiformes	XP_016521686.1
INS	*Chrysemys picta bellii* (Western painted turtle)	Chordata	Reptilia	Testudines	XP_005312438.1
TCAP1	*Xenopus laevis* (African clawed frog)	Chordata	Amphibia	Anura	XP_017951867.1
TCAP1	*Callorhinchus milii* (Elelphant shark)	Chordata	Chondrichthyes	Chimaeriformes	XP_007893009.1
TCAP	*Caenorhabditis elegans* (Roundworm)	Nematoda	Secernentea	Rhabditida	NM_171175
TCAP	*Drosophila melanogaster* (Fruit fly)	Arthropoda	Insecta	Diptera	NP_001097661
TCAP1	*Mus musculus* (House mouse)	Chordata	Mammalia	Rodentia	NP_035985.2
TCAP1	*Danio rerio* (Zebrafish)	Chordata	Actinopterygii	Cypriniformes	XP_691552.5
TCAP1	*Columba livia* (Rock pigeon)	Chordata	Aves	Columbiformes	EMC88689.1
TCAP2	*Xenopus laevis* (African clawed frog)	Chordata	Amphibia	Anura	XP_012815129.1
TCAP2	*Callorhinchus milii* (Elelphant shark)	Chordata	Chondrichthyes	Chimaeriformes	XP_007900206.1
TCAP2	*Mus musculus* (House mouse)	Chordata	Mammalia	Rodentia	NP_035986.3
TCAP2	*Danio rerio* (Zebrafish)	Chordata	Actinopterygii	Cypriniformes	XP_017208443.1
TCAP2	*Columba livia* (Rock pigeon)	Chordata	Aves	Columbiformes	EMC78205.1
TCAP3	*Xenopus laevis* (African clawed frog)	Chordata	Amphibia	Anura	NP_001096158
TCAP3	*Callorhinchus milii* (Elelphant shark)	Chordata	Chondrichthyes	Chimaeriformes	XP_007894102.1
TCAP3	*Strongylocentrotus purpuratus* (Sea urchin)	Echinodermata	Echinoidea	Echinoidea	XM_001180001
TCAP3	*Mus musculus* (House mouse)	Chordata	Mammalia	Rodentia	NP_035987.3
TCAP3	*Danio rerio* (Zebrafish)	Chordata	Actinopterygii	Cypriniformes	NP_571043.1
TCAP3	*Columba livia* (Rock pigeon)	Chordata	Aves	Columbiformes	XP_005505621.1
TCAP4	*Xenopus laevis* (African clawed frog)	Chordata	Amphibia	Anura	NP_001096158.1
TCAP4	*Callorhinchus milii* (Elelphant shark)	Chordata	Chondrichthyes	Chimaeriformes	XP_007900970.1
TCAP4	*Mus musculus* (House mouse)	Chordata	Mammalia	Rodentia	NP_001297689.1
TCAP4	*Danio rerio* (Zebrafish)	Chordata	Actinopterygii	Cypriniformes	NP_571044.2
TCAP4	*Columba livia* (Rock pigeon)	Chordata	Aves	Columbiformes	XP_005500626.1

### Sequence Alignments

Peptide sequences were aligned using the MUSCLE algorithm (39). The alignment was examined, reviewed for duplicate sequences using pairwise distances (*d* = 0.0 was identical) and excess sequence was cut at both 5′ and 3′ ends, as these fragments did not contribute to the alignment. Modifications to the alignment were made to ensure that the characteristic residue motifs were conserved. This included highly conserved cysteine (C), tryptophan (W), arginine (R), and lysine (K) residues throughout as well as motifs characteristic of each family. For the CRF family this was the 5′ leucine (L), serine (S), and the 3′ asparagine (N) motif that is conserved throughout the entire family, the “TCV” or “TCXV” motif that is conserved among the calcitonin family and the “PELAD” motif that is conserved among the TCAP family.

### Phylogenetic Analysis

Phylogenetic tree construction and statistical analyses were carried out in MEGA 6.0 (38). A multi-step approach was undertaken in order to understand the relationship of each family relative to TCAP prior to conducting a comprehensive analysis of all of the families.

#### Maximum Likelihood (ML) Method

The amino acid substitution model and the rate among sites were both chosen based on the model that resulted in the greatest log likelihood, the lowest Akaike Information Criterion (AIC) and the lowest Bayesian Information Criterion (BIC), parameters calculated by MEGA 6.0. To ensure the most accurate analysis, these parameters were calculated for each constructed tree. The model that maximized the log likelihood was used for analysis. A partial deletion of sequences with too many gaps/missing data was applied with a cutoff of 95%, so sites that were not found in at least 95% of sequences were not used toward the analysis. The applied heuristic method was Nearest-Neighbor Interchange (NNI), so the initial trees were obtained using the NJ method to a matrix of pairwise distances estimated using a JTT model. Reliability of the tree was tested using 1,000 bootstrap replicates.

#### Pre-propeptide and Mature Peptide Analysis

Two sets of analyses were performed. The first involved Secretin superfamily pre-propeptides, which are composed of a signal, cryptic, and mature peptide and TCAP propeptides, as TCAP does not possess a signal peptide. Given the functional importance, bioactivity, and high level of conservation throughout evolution, a second separate analysis was performed on mature peptides of both Secretin superfamily and TCAP family members.

For analysis involving Secretin superfamily pre-propeptides and TCAP family propeptides, a total of 181 amino acid sequences were used, with a total of 44 positions in the final dataset after all positions with <95% site coverage were eliminated.

#### Mature Peptide Analysis

For analysis involving Secretin superfamily mature peptides and TCAP mature peptides, a multi-step analysis was undertaken in order to elucidate the relationships of each family with respect to one another and TCAP. As insulin has a tertiary structure where the peptide folds and the two mature chains are connected by sets of disulfide bonds from the cysteine residues (40), the mature peptide had to be divided into A and B chains for the purpose of this analysis. Due to the high sequence conservation of NPY that may have resulted in the odd placement of the NPY reference group in the pre-propeptide analysis and given that the NPY mature peptide is even so more highly conserved, it was not included as a reference group in the analysis of mature peptides. For insulin and the calcitonin family, analysis involved 72 amino acid sequences, with a total of 14 positions in the final dataset. For insulin, calcitonin, and TCAP, analysis involved 95 amino acid sequences, with a total of 14 positions in the data set. For insulin, calcitonin, CRF and TCAP families, analysis involved 135 amino acid sequences leaving 12 positions in the final data set. Lastly, for insulin, calcitonin, CRF, secretin, and TCAP families, analysis included 179 amino acid sequences leaving 15 positions in the final dataset.

## Results

### Sequence Analysis of TCAP Paralogs and Orthologs

**Figure 1:**
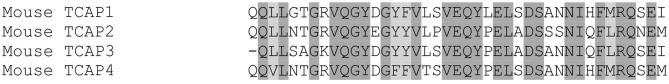
Multiple sequence alignment of the TCAP family of peptides in mouse. The mature peptide sequences were aligned using MUSCLE (MUltiple Sequence Comparison by Log-Expectation). Dark gray boxes indicate amino acid identity and light gray boxes indicate a functional replacement.

**Figure 2:**
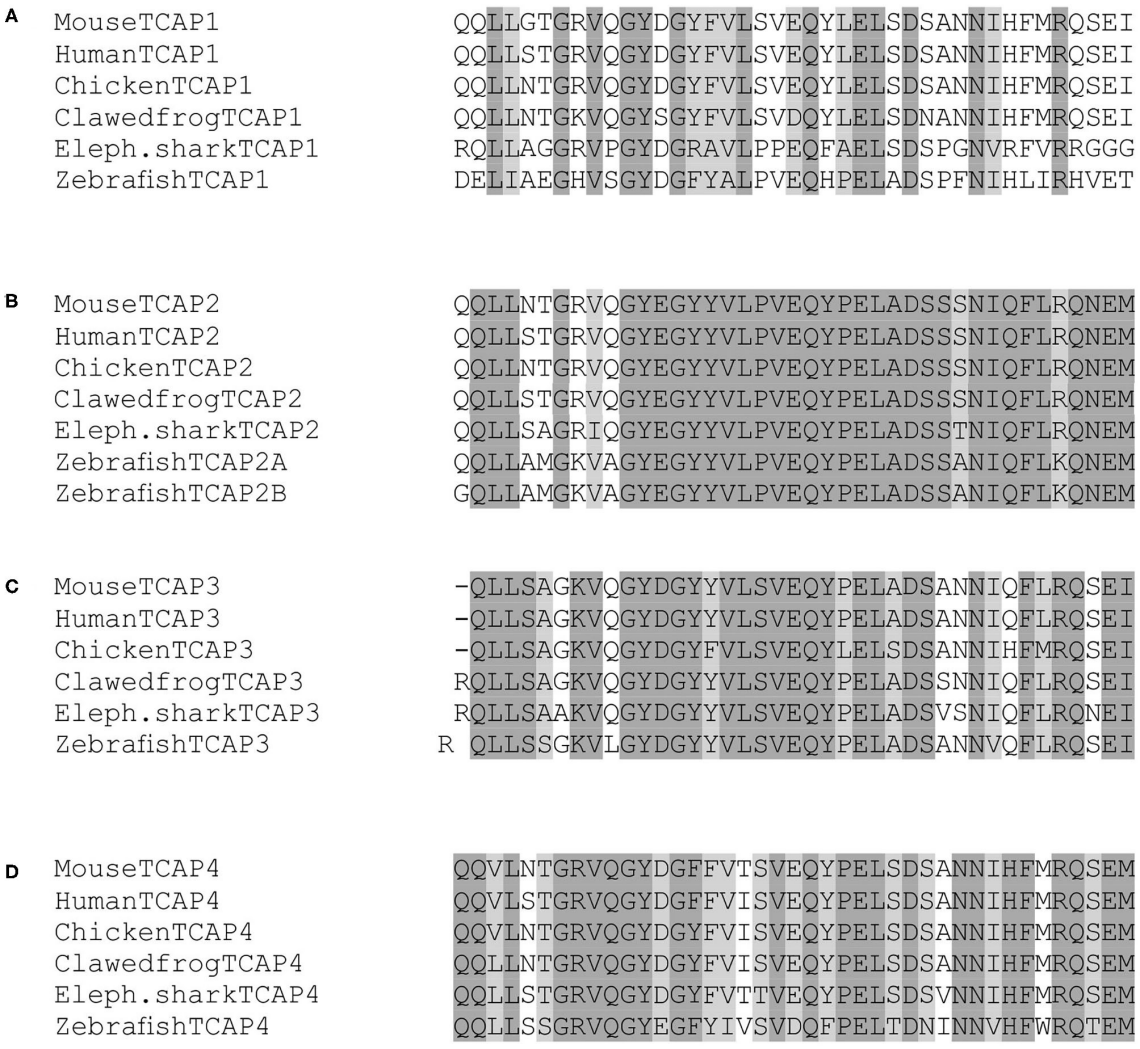
Multiple sequence alignment TCAP family members among various species. **(A)** TCAP-1; **(B)** TCAP-2; **(C)** TCAP-3; **(D)** TCAP-4. The mature peptide sequences were aligned using MUSCLE (Multiple Sequence Comparison by Log-Expectation). Dark gray boxes indicate amino acid identity and light gray boxes indicate a functional replacement.

TCAP paralogs, those that diverged as a result of a genome duplication event, demonstrated a high degree of conservation (Figure 1). When TCAP 1–4 are aligned in mouse, residues Q2, L4, G7, V9, Q10, G11, Y12, G14, V17, V20, E21, Q22, Y23, E25, L26, D28, S29, N32, I33, F35, R37, Q38, and E40 are all conserved among the four paralogs (Figure 1). Similarly, TCAP orthologs, those that arose as a result of a species divergence, demonstrate a high degree of conservation among vertebrates (Figure 2). When mammalian, bird, amphibian, and fish TCAP 1-4 sequences are aligned residues L3, G7, V9, G11, Y12, G14, L18, Q22, E25, L26, D28, N32, R37 are conserved among TCAP-1 orthologs (Figure 2A). Among TCAP-2 orthologs, residues Q2, L3, L4, G7, G11, Y12, E13, G14, Y15, Y16, V17, L18, P19, V20, E21, Q22, Y23, P24, E25, L26, A27, D28, S29, S30, N32, I33, Q34, F35, L36, Q38, N39, E40, M41 are conserved (Figure 2B). Among TCAP-3 orthologs, Q2, L3, L4, S5, K8, V9, G11, Y12, D13, G14, Y15, V17, L18, S19, V20, E21, Q22, Y23, E25, L26, D28, S29, N32, F35, R37, Q38, E40, I41 are conserved (Figure 2C). Lastly, among TCAP-4 orthologs, Q1, Q2, L4, G7, R8, V9, Q10, G11, Y12, G14, F15, V20, Q22, P24, E25, L26, D28, N31, N32, H34, F35, R37, Q38, E40, M41. Overall, TCAP-2 orthologs (Figure 2B) are the most highly conserved and TCAP-1 orthologs (Figure 2A) are the least highly conserved. Also, a characteristic “PELAD” motif at positions 24–28 from the N-terminus is conserved among the TCAP paralogs and orthologs. The high level of conservation of the “PELAD” motif suggests that it possesses a functional attribute, such as a receptor binding or activation site (27). This family of peptides contains the “PELAD” motif at residues 24–28 from the N-terminus. Therefore, both TCAP orthologs and paralogs demonstrate a high degree of conservation among vertebrates.

### Evolutionary Analysis of Pre-propeptides and Mature Peptides of Secretin Superfamily and TCAP Family Members

**Figure 3:**
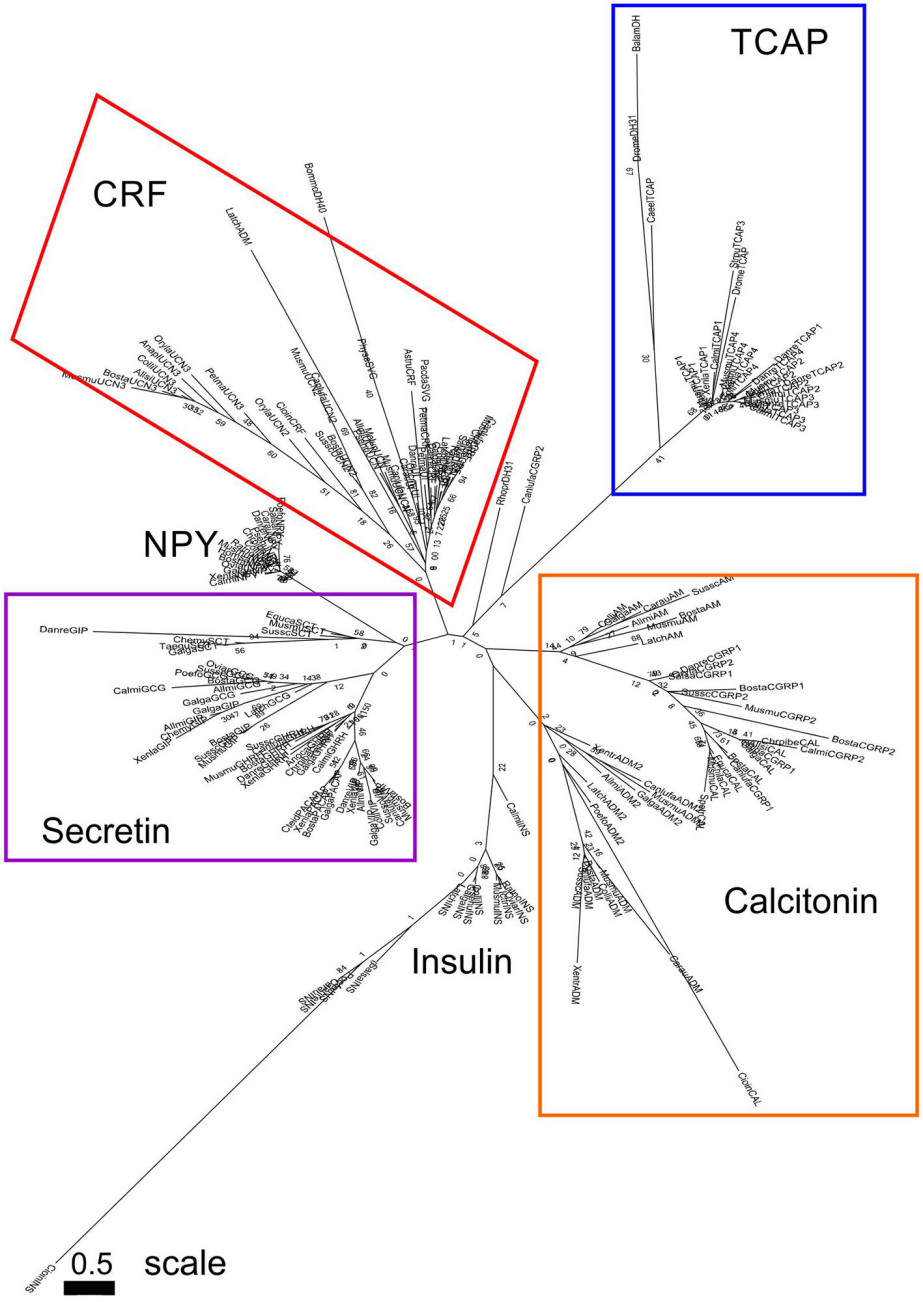
Phylogenetic analysis of CRF, calcitonin, insulin, and secretin family pre-propeptides with TCAP propeptides (rooted to TCAP). Each family is highlighted with a different color: CRF (red), calcitonin (orange), secretin (purple), TCAP (blue). Analysis was conducted using the maximum likelihood method based on the JTT+G matrix-based model (lnL = −11224.5064; +G, parameter = 1.3976) (41). Initial trees for the heuristic search were obtained by applying the NJ method to a matrix of pairwise distances estimated using a JTT model. Branch lengths represent the number of substitutions per site, with the tree shown to scale. Bootstrap analysis involved 1,000 replicates. CRF family: CRF, corticotropin-releasing factor; TCN, teleocortin; UCN, urocortin; UCN2, urocortin 2; UCN3, urocortin 3; UI, urotensin; SVG, sauvagine; DH, diuretic hormone; Calcitonin family: CALC, calcitonin; CGRP1, calcitonin-gene-related peptide 1; CGRP2, calcitonin-gene-related peptide 2; AM, amylin; ADM, adrenomedullin; ADM2, adrenomedullin 2; Secretin family: SCT, secretin; GHRH, growth hormone releasing hormone; GIP, gastric inhibitory peptide; GCG, glucagon; PACAP, pituitary adenylate cyclase-activating peptide; VIP, vasoactive intestinal peptide; Reference groups: NPY, neuropeptide Y; INS, insulin; Outgroup: TCAP, teneurin C-terminal associated peptide. The scale bar indicates the level of magnification for the tree.

Phylogenetic analysis of CRF, calcitonin, and secretin pre-propeptide families and TCAP family propeptides revealed that each family formed a distinct group. TCAP, CRF, and secretin families form distinct clades and insulin forms a sister group with the calcitonin family (Figure 3). Also, CRF and calcitonin are closely related sister lineages and they, in turn, form a sister lineage to the secretin family. TCAP, the putative progenitor, is most distantly related to these families relative to their relationships to one another.

**Figure 4:**
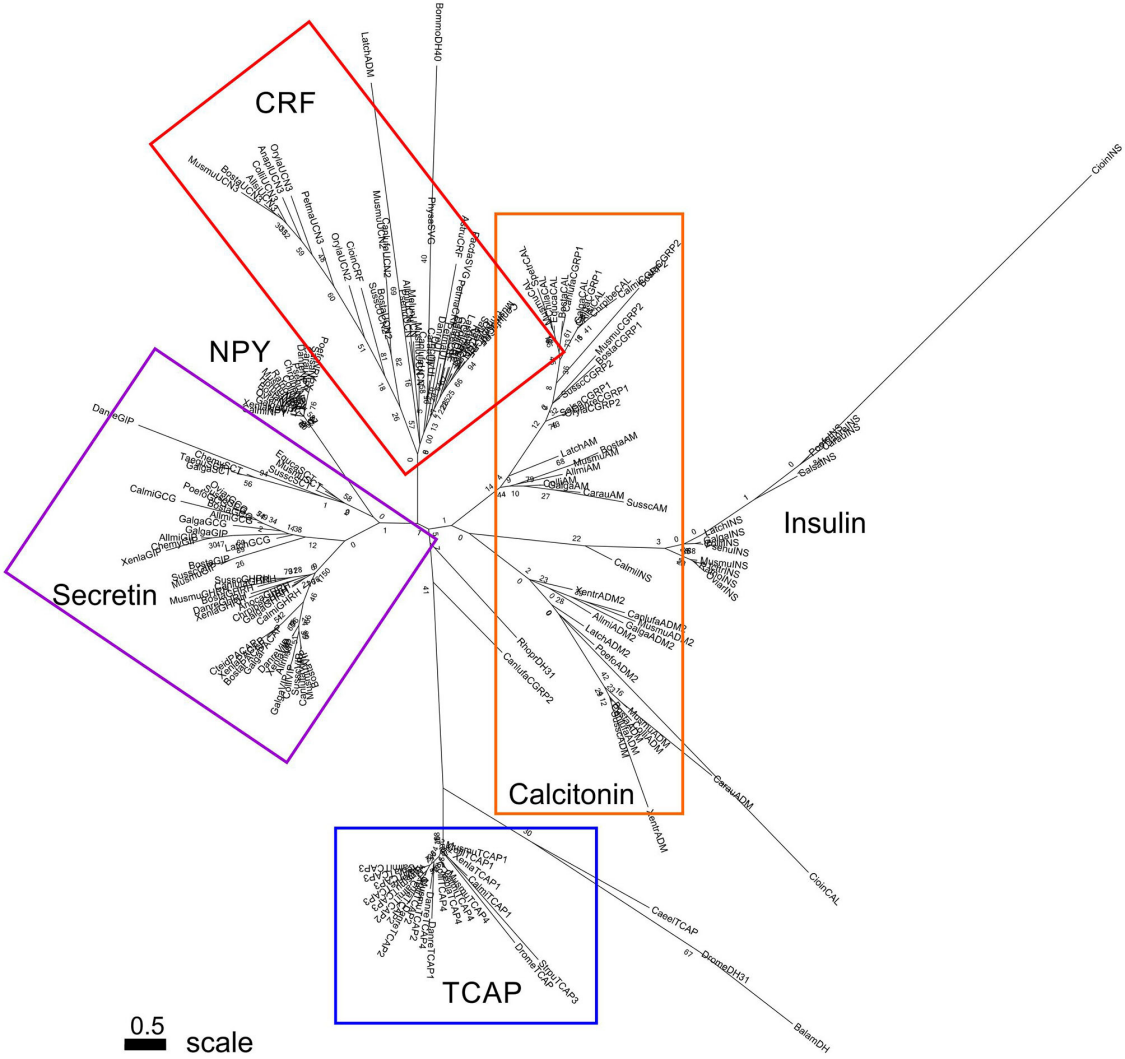
Phylogenetic analysis of insulin, calcitonin and TCAP mature peptides (rooted to TCAP). Each family is highlighted with a different color: calcitonin (orange), insulin (green), TCAP (blue). Analysis was conducted using the maximum likelihood method based on the JTT matrix-based model (lnL = −919.2846; +G, parameter = 6.6766) (41) Initial trees for the heuristic search were obtained by applying the NJ method to a matrix of pairwise distances estimated using a JTT model. Branch lengths represent the number of substitutions per site, with the tree shown to scale. Bootstrap analysis involved 1,000 replicates. Calcitonin family: CALC, calcitonin; CGRP1, calcitonin-gene-related peptide 1; CGRP2, calcitonin-gene-related peptide 2; AM, amylin; ADM, adrenomedullin; ADM2, adrenomedullin 2; Insulin: INSa, insulin A chain; INSb, insulin B chain; Outgroup: TCAP, teneurin C-terminal associated peptide. The scale bar indicates the level of magnification for the tree.

**Figure 5:**
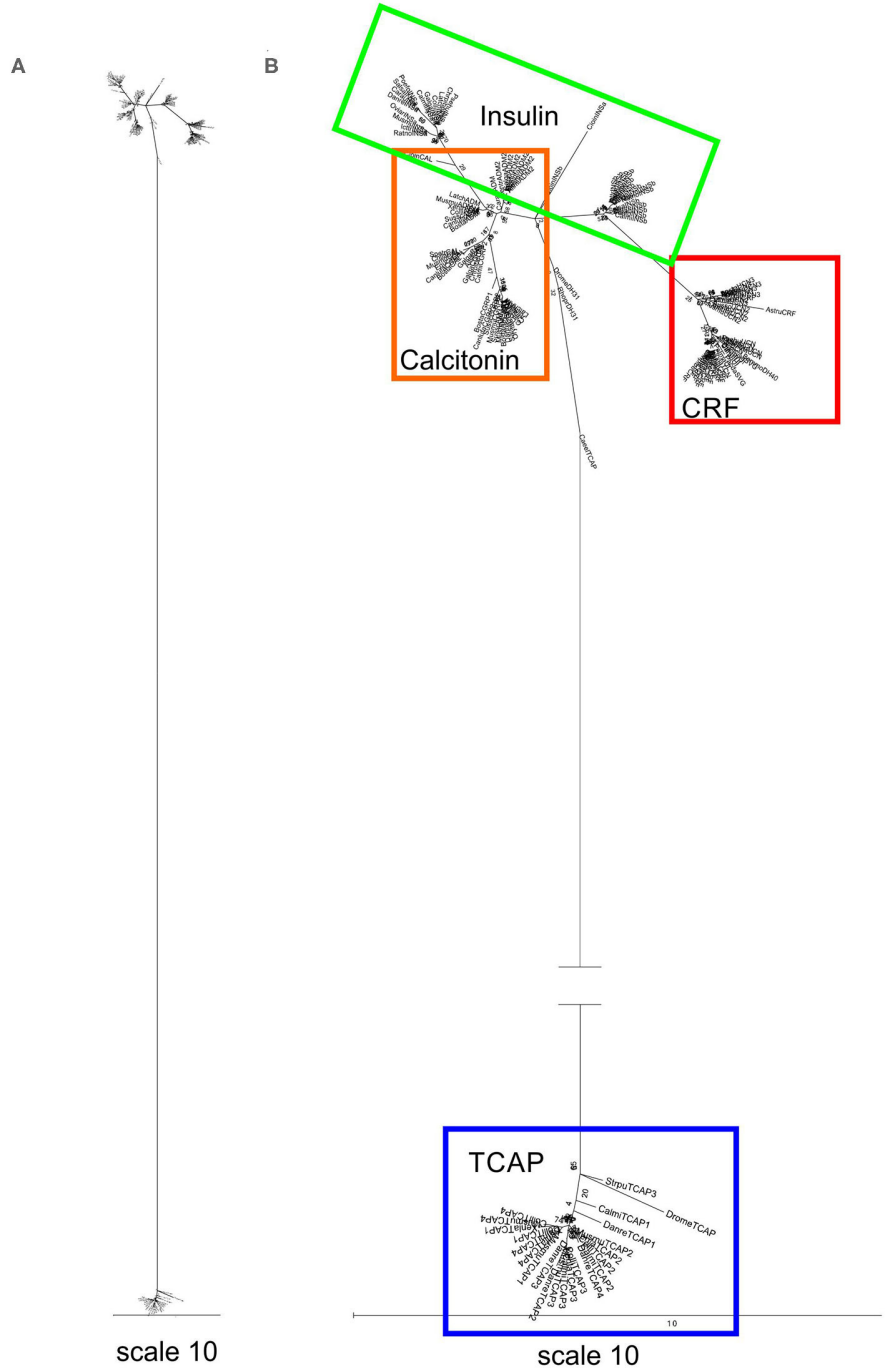
Phylogenetic analysis of insulin, calcitonin, CRF and TCAP mature peptides (rooted to TCAP). The trees are represented as **(A)** original tree with the appropriate scale **(B)** magnified and rooted to TCAP. Each family is highlighted with a different color: CRF (red), calcitonin (orange), insulin (green), TCAP (blue). Analysis was conducted using the maximum likelihood method based on the Dayhoff matrix-based model (lnL = −1019.5552; +G, parameter = 6.6766) (41). Initial trees for the heuristic search were obtained by applying the NJ method to a matrix of pairwise distances estimated using a JTT model. Branch lengths represent the number of substitutions per site, with the tree shown to scale. Bootstrap analysis involved 1,000 replicates. Calcitonin family: CALC, calcitonin; CGRP1, calcitonin-gene-related peptide 1; CGRP2, calcitonin-gene-related peptide 2; AM, amylin; ADM, adrenomedullin; ADM2, adrenomedullin 2; Insulin: INSa, insulin A chain; INSb, insulin B chain; CRF family: CRF, corticotropin-releasing factor; TCN, teleocortin; UCN, urocortin; UCN2, urocortin 2; UCN3, urocortin 3; UI, urotensin; SVG, sauvagine; DH, diuretic hormone; Outgroup: TCAP, teneurin C-terminal associated peptide. The scale bar indicates the level of magnification for the tree.

**Figure 6:**
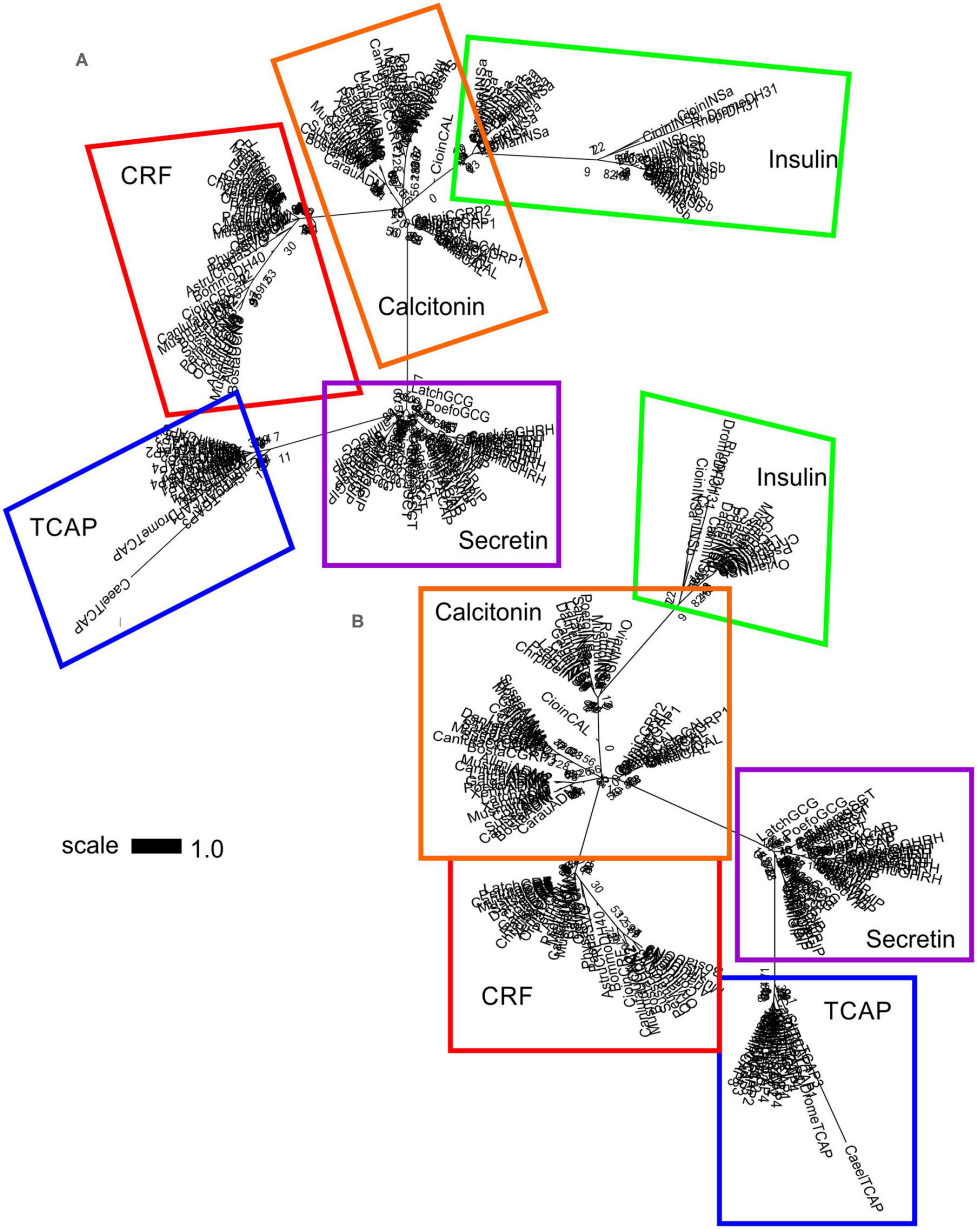
Phylogenetic analysis of insulin, calcitonin, CRF, secretin, and TCAP mature peptides. The trees are represented as **(A)** unrooted and **(B)** rooted to TCAP. Each family is highlighted with a different color: CRF (red), calcitonin (orange), insulin (green), secretin (purple), and TCAP (blue). Analysis was conducted using the maximum likelihood method based on the Whelan and Goldman model (lnL = −1781.0007; +G, parameter = 23.5912) (41). Initial trees for the heuristic search were obtained by applying the NJ method to a matrix of pairwise distances estimated using a JTT model. Branch lengths represent the number of substitutions per site, with the tree shown to scale. Bootstrap analysis involved 1,000 replicates. Calcitonin family: CALC, calcitonin; CGRP1, calcitonin-gene-related peptide 1; CGRP2, calcitonin-gene-related peptide 2; AM, amylin; ADM, adrenomedullin; ADM2, adrenomedullin 2; INSa, insulin A chain; INSb, insulin B chain; CRF family: CRF, corticotropin-releasing factor; TCN, teleocortin; UCN, urocortin; UCN2, urocortin 2; UCN3, urocortin 3; UI, urotensin; SVG, sauvagine; DH, diuretic hormone; Secretin family: SCT, secretin; GHRH, growth hormone releasing hormone; GIP, gastric inhibitory peptide; GCG, glucagon; PACAP, pituitary adenylate cyclase-activating peptide; VIP, vasoactive intestinal peptide; Outgroup: TCAP, teneurin C-terminal associated peptide. The scale bar indicates the level of magnification for the tree.

A separate analysis was performed with mature peptide sequences of the Secretin superfamily and TCAP mature peptides due to their high conservation and functional importance throughout evolution. Phylogenetic analysis of calcitonin mature peptides, insulin A and B mature chains and TCAP demonstrated that calcitonin and insulin families are sister lineages (Figure 4). Insulin A chains were more closely related to the calcitonin family than insulin B chains (Figure 4). Phylogenetic analysis of calcitonin, insulin A and B chains, CRF, and TCAP mature peptides confirmed that calcitonin and insulin families were sister lineages and that CRF formed a separate group to these two families (Figure 5). Lastly, phylogenetic analysis of calcitonin, insulin A and B chains, CRF, secretin, and TCAP mature peptides revealed that calcitonin and insulin families were sister lineages and that both CRF and secretin formed separate groups from these two families (Figure 6). Therefore, the multi-step mature peptide analysis confirmed that insulin and calcitonin are sister lineages, that form distinct groups from CRF and secretin families and in turn, that the TCAP family is a distinct clade from Secretin superfamily members.

## Discussion

In this study, the TCAP family is presented as a putative progenitor of the Secretin superfamily of ligands for the first time. The evolutionary relationships among the receptors of these peptides are well-established (18, 19). However, the relationships among members of the Secretin superfamily of ligands as well as a progenitor for this family of peptides have not been elucidated. We considered TCAP as a putative progenitor of the Secretin superfamily for the following reasons. First, evolutionary relationships among the receptors of these ligands demonstrate that Secretin GPCRs derived from Adhesion GPCRs (19) and as TCAP-1 binds to LPHN, an Adhesion GPCR with a HBD characteristic of Secretin GPCRs (17). It is possible that a similar course of evolution occurred for the ligands. Second, the sequence similarity that TCAP shares with CRF and calcitonin (27), both Secretin superfamily members whose receptors are the most closely related to Adhesion GPCRs, suggests that these peptides may have evolved from TCAP, a candidate progenitor peptide.

The teneurin-TCAP system is well-established as being evolutionarily ancient. Evidence suggests that this system arose before the Metazoa evolved about 1 billion years ago and prior to the emergence of the Secretin superfamily that arose around the time of the protostome-deuterostome divergence, about 600 million years ago. As a result, although the TCAP sequence shows some amino acid similarity with the Secretin superfamily, there are a number of differences indicating that the two lineages are evolutionarily divergent. Indeed, we could not determine any significant binding or activation capacity of TCAP with any members of the Secretin GPCRs [(11, 34); Lovejoy, unpublished observations]. In contrast, TCAP binds to the latrophilin HBD and activates this receptor [(30); Reid et al., submitted]. As proposed by Zhang et al. (33), the teneurin-TCAP system likely evolved from a polymorphic proteinaceous toxin (PPT) gene that arose as a result of a HGT event from a prokaryote to a choanoflagellate, a primitive unicellular organism. Importantly, the teneurin gene has been identified in the choanoflagellate, *Monosiga brevicollis* (32). Choanoflagellates are thought to be a progenitor to the Metazoans (42). This supports the hypothesis that a choanoflagellate may have engulfed a prokaryote containing the PPT gene, which became integrated into its genome and lost its toxic role over time (32, 33). With respect to structural evidence, the teneurins share characteristics of PPTs: the same type II orientation, rearrangement hotspot (RHS) domains and close similarity to the C-terminal domain to the histidine-asparagine-histidine (HNH) bacterial toxin of the glycine-histidine-histidine (GHH) clade (33, 43). The GHH domain may be an ancestor of TCAP that lost its toxic role and functioned as an intracellular signaling molecule (33). Additionally, the C-terminal region of the *M. brevicollis* teneurin protein contains tyrosine-aspartate (YD) repeats characteristic of proteobacteria and most of the extracellular domain is encoded on one large 6,829 base pair exon characteristic of prokaryotic genomes and of HGT (32). Therefore, evidence suggests that the teneurin-TCAP system is ancient as it evolved as a result of a HGT event prior to the emergence of the Metazoa.

Moreover, with respect to the course of evolution of the receptors, evidence demonstrates that Adhesion GPCRs evolved prior to Secretin GPCRs and that Secretin GPCRs are derived from Adhesion GPCRs. Adhesion GPCR genes have been identified in the genome of amphioxus, *Branchiostoma floridae*, the choanoflagellate, *M. brevicollis*, and the sea anemone, *Nematostella vectensis* (18), meaning that these lineages were present prior to the protostome-deuterostome divergence. On the other hand, Secretin GPCRs have not been identified in these species and therefore, receptor lineages of the Secretin superfamily likely expanded and radiated around the time of the bifurcation of protostomes and deuterostomes. Also, Nordström et al. (18) demonstrated Secretin GPCRs evolved from Adhesion GCPRs using phylogenetic analysis. Therefore, evidence that the teneurin-TCAP system arose prior to the emergence of the Metazoa as well as the characterization of Adhesion GPCRs but not Secretin GPCRs prior to the protostome-deuterostome divergence suggests that the teneurin-TCAP system predates members of the Secretin superfamily. We suggest that if the ligands for these receptors underwent a similar course in evolution, the TCAP family may be a putative progenitor to the Secretin superfamily.

In light of the evidence to suggest that the teneurin-TCAP system evolved prior to the emergence of the Metazoa, the previously established relationship that Secretin GPCRs derived from Adhesion GPCRs [(Nordstom et al., 2009); (19)], the evidence that TCAP binds to LPHN, an Adhesion GPCR with a HBD characteristic of Secretin GPCRs (17) and given the sequence similarity that TCAP shares with Secretin superfamily members, CRF, and calcitonin (27), a phylogenetic investigation using TCAP as a putative progenitor of the Secretin superfamily was undertaken. A putative progenitor of the Secretin superfamily of ligands has not been previously established. Sequence analysis of TCAP family members demonstrated a highly conserved peptide and phylogenetic analysis of the Secretin superfamily in relation to TCAP as a putative progenitor revealed relationships among Secretin superfamily members. Calcitonin and insulin families are sister lineages and they are much more closely related to one another than was previously thought. Also, calcitonin and insulin are sister lineages that form distinct lineages to CRF and secretin families. Therefore, placing TCAP as an ancestor of the Secretin superfamily allowed a novel interpretation of evolutionary relationships among Secretin superfamily members.

### Sequence Analysis of TCAP Paralogs and Orthologs

Sequence analysis of both TCAP paralogs and orthologs revealed that this family of peptides is highly conserved. The presence of a conserved “PELAD” motif among TCAP orthologs and paralogs, suggests that it may possess a functional attribute, such as a receptor-binding or activation site (27). Also, some characteristic amino acids are retained throughout orthologs and paralogs. Arginine (R) and lysine (K) residues are retained in some parts of the mature peptide and they are often characteristic of the presence of cleavage sites. Glycine (G) and proline (P) are also highly conserved and these amino acids have a tendency to be retained as their secondary structure can break the α-helical structure of peptides. A peptide system with such a large amount of conservation is indicative of great functional importance that may have been selected for. Therefore, the high sequence conservation among TCAP orthologs and paralogs suggests that this peptide system is evolutionarily ancient and may have been strongly selected for throughout evolutionary time.

### Evolutionary Analysis of Pre-propeptides and Mature Peptides of Secretin Superfamily and TCAP Family Members

Phylogenetic analysis of Secretin superfamily pre-propeptides (composed of the signal, cryptic, and mature peptide) and TCAP family pro-peptides (composed of the cryptic and mature peptide) was undertaken in order to elucidate the relationships among these peptides. Analysis revealed that calcitonin, CRF, secretin, and TCAP families formed distinct groups. Despite being chosen to serve as a reference group because it binds to a tyrosine kinase receptor and not a GPCR, insulin formed a group with calcitonin, suggesting that they may be sister lineages (Figure 3). The close relationship between calcitonin and insulin has previously been explored where Wimalawansa (44) suggested that insulin and calcitonin families are closely related. This is supported by phylogenetic analysis of the pre-propeptides and suggests that insulin and calcitonin are sister lineages. When the tree was rooted to TCAP (Figure 3), to establish the assumption that TCAP is the ancestor, CRF, calcitonin, and secretin families formed distinct groups. This evolutionary analysis suggests that the secretin family forms a separate clade that is a sister to CRF and calcitonin families, which, in turn, are sisters to one another. This is consistent with what has been observed with respect to Secretin GPCR evolution, where CRF and calcitonin receptors share the greatest amount of sequence similarity among Secretin GPCRs (17). Therefore, it is possible that a similar evolutionary scheme occurred with respect to the ligands. Thus, analysis of Secretin superfamily pre-propeptides with TCAP propeptides suggests that insulin and calcitonin are closely related sister lineages, that calcitonin-insulin and CRF lineages are closely related and that calcitonin-insulin and CRF form a distinct sister lineage to the secretin family.

Subsequently, phylogenetic analysis was performed with the mature peptides of Secretin superfamily members and the TCAP family. The analysis of TCAP family mature peptide sequences with calcitonin and insulin mature sequences (Figure 4) demonstrated that insulin A chains were closely related to mature calcitonin peptides. This suggests that the insulin A mature chain is more closely related to the calcitonin family than the insulin B mature chain, which is different from what was previously suggested by Wimalawansa (44). Subsequent analyses involving CRF, calcitonin, insulin, and TCAP mature peptides (Figure 5) as well as secretin, CRF, calcitonin, insulin, and TCAP mature peptides (Figure 6) confirmed that the insulin A chain was more closely related to the calcitonin family than the insulin B chain. Taken together, insulin and calcitonin are closely related sister groups, which was also observed with the pre-propeptide analysis (Figure 3). Moreover, with respect to relationships among Secretin superfamily members, calcitonin-insulin, and CRF families are more closely related to one another than they are to secretin or TCAP, which is supported by the evolutionary scheme of their receptors, which also appear to be very closely related. Finally, secretin forms a sister lineage to a lineage that comprises both calcitonin-insulin and CRF families. This is consistent with what was observed for analysis of the pre-propeptides (Figure 3).

Considering the evidence with respect to the ancestral origin of the teneurin-TCAP system and in light of the findings presented here, it is possible to present two hypotheses for the evolutionary scheme of these peptides. The first suggests that an ancient TCAP-like peptide may have been the ancestor of the Secretin superfamily and that it evolved prior to the emergence of CRF, calcitonin, and secretin families. This is supported by the identification of TCAP in organisms prior to the protostome-deuterostome divergence, where as members of the Secretin superfamily have not been identified this early in evolution (31, 32, 34). The possibility of a second hypothesis, suggesting that the Secretin superfamily forms a parallel lineage to extant TCAP and that these two lineages evolved from a proto-CRF-calcitonin-secretin-TCAP ancestor that was related to all of these families, cannot be discounted. Due to sequence availability, phylogenetic analysis was performed using extant Secretin superfamily and TCAP sequences. As a result, both of these hypotheses are plausible. Future analysis should be undertaken in order to further investigate whether TCAP is a progenitor of the Secretin superfamily of ligands.

## Conclusions

Taken together, phylogenetic analysis of members of the Secretin superfamily using TCAP as a putative progenitor demonstrated relationships among Secretin superfamily members. First, calcitonin formed a closely related sister lineage to insulin, particularly the insulin A chain with respect to mature peptides, but this was also observed with the pre-propeptides. Also, calcitonin-insulin and CRF families are more closely related to one another than they are to secretin or TCAP, which is supported by the evolutionary scheme of their receptors. Finally, secretin forms a sister lineage to a group that comprises both calcitonin-insulin and CRF. Therefore, given evidence that the teneurin-TCAP system arose as a result of a HGT event prior to the emergence of the Metazoa, as well as the previously established structural similarity of TCAP to calcitonin and CRF, members of the Secretin superfamily, the presented phylogenetic analysis allowed for the elucidation of relationships among members of the Secretin superfamily. To conclude, this is the first time that relationships among this family of peptides were resolved and because a progenitor peptide for the Secretin superfamily has not been elucidated, we present TCAP as a candidate progenitor.

## Data Availability Statement

The raw data supporting the conclusions of this article will be made available by the authors, without undue reservation.

## Author Contributions

OM performed all analyses and completed the first draft of the paper. BC and NL provided technical guidance on the construction of the phylogenetic tree. DL oversaw the research program and completed the final draft of the manuscript. All authors contributed to the article and approved the submitted version.

## Conflict of Interest

The authors declare that the research was conducted in the absence of any commercial or financial relationships that could be construed as a potential conflict of interest.
